# Erythropoietin Slows Photoreceptor Cell Death in a Mouse Model of Autosomal Dominant Retinitis Pigmentosa

**DOI:** 10.1371/journal.pone.0157411

**Published:** 2016-06-14

**Authors:** Tonia S. Rex, Lorraine Kasmala, Wesley S. Bond, Ana M. de Lucas Cerrillo, Kristi Wynn, Alfred S. Lewin

**Affiliations:** 1 Vanderbilt Eye Institute, Vanderbilt Brain Institute, Vanderbilt University, Nashville, TN 37232, United States of America; 2 Department of Molecular Genetics & Microbiology, University of Florida, Gainesville, FL 32608, United States of America; University of Cologne, GERMANY

## Abstract

**Purpose:**

To test the efficacy of systemic gene delivery of a mutant form of erythropoietin (EPO-R76E) that has attenuated erythropoietic activity, in a mouse model of autosomal dominant retinitis pigmentosa.

**Methods:**

Ten-day old mice carrying one copy of human rhodopsin with the P23H mutation and both copies of wild-type mouse rhodopsin (hP23H *RHO*^+/-^,m*RHO*^+/+^) were injected into the quadriceps with recombinant adeno-associated virus (rAAV) carrying either enhanced green fluorescent protein (eGFP) or EpoR76E. Visual function (electroretinogram) and retina structure (optical coherence tomography, histology, and immunohistochemistry) were assessed at 7 and 12 months of age.

**Results:**

The outer nuclear layer thickness decreased over time at a slower rate in rAAV.EpoR76E treated as compared to the rAAV.eGFP injected mice. There was a statistically significant preservation of the electroretinogram at 7, but not 12 months of age.

**Conclusions:**

Systemic EPO-R76E slows death of the photoreceptors and vision loss in hP23H *RHO*^+/-^,m*RHO*^+/+^ mice. Treatment with EPO-R76E may widen the therapeutic window for retinal degeneration patients by increasing the number of viable cells. Future studies might investigate if co-treatment with EPO-R76E and gene replacement therapy is more effective than gene replacement therapy alone.

## Introduction

Retinitis pigmentosa (RP) is a group of inherited retinal degenerations that affects over 1.5 million people world-wide. Over 200 causative genes have been identified, but the most common is rhodopsin, which is responsible for 40% of all known cases of RP.[1–3] In particular, the P23H mutation of rhodopsin is the most common cause of autosomal dominant retinitis pigmentosa (ADRP) in North America, as it is responsible for 12% of all known cases.[3–5] In this form of RP, the mutant rhodopsin is misfolded, which may lead to endoplasmic reticulum stress and, ultimately, rod cell death (for review see [6]). However, in P23H knock-in mice, the IRE1 pathway and not the pro-apoptotic PERK pathway is activated.[7] Further, virus-mediated gene delivery of wild-type rhodopsin into hP23H *RHO*^+/-^,m*RHO*^+/+^ mice without removal of the mutant form was beneficial based on both histology and visual function.[8] Degeneration of the photoreceptors leads to neuroinflammation, including reactivity of both microglial and macroglial cells.[9, 10] Therefore, a neuroprotective approach to normalize the environment and block photoreceptor death until gene-specific therapies can be developed, or in conjunction with gene-specific therapies to provide synergistic benefit, is attractive.

Erythropoietin (EPO) is a secreted cytokine that is FDA approved and used in the clinic for the treatment of anemia. It is also an effective neuroprotective agent in numerous animal models and is currently being tested in clinical trials for this purpose (ClinicalTrials.gov; for review see[11]). Both EPO and its receptor are found at low levels in the brain and retina.[12] EPO can block apoptotic pathways directly through increasing levels of Bcl-xL and modulating levels of intracellular calcium through activation of phospholipase Cγ.[13, 14] Particularly relevant to ADRP due to P23H rhodopsin mutants, EPO can decrease levels of CHOP and other ER stress-induced proteins in other models.[15] It can also protect neurons indirectly by increasing levels of anti-oxidant enzymes through activation of the antioxidant response element and decreasing neuroinflammation including decreasing glial reactivity.[13, 16]

Treatment with EPO or EPO-R76E, a form of EPO that has attenuated erythropoietic activity, protect the retinal ganglion cells in models of glaucoma and protects the photoreceptors in the light-induced and *retinal degeneration slow* (Rds) models of retinal degeneration and in trauma.[17–27] The goal of this study was to determine if recombinant adeno-associated (rAAV) mediated delivery of EPO-R76E is effective in slowing the rate of photoreceptor degeneration in the hP23H *RHO*^+/-^,m*RHO*^+/+^ mouse model of ADRP.

## Materials and Methods

### Mice

The hP23H *RHO*^+/-^,m*RHO*^+/+^ mice were provided by the University of Florida.[8] Mice were housed in normal cyclic light conditions (12 hours light: 12 hours dark), in the middle to lower part of the rack. Littermate controls were used for all studies and all correctly genotyped mice in a litter were used. Euthanasia was performed by anesthetic overdose and perfusion. All procedures and housing were in accordance with the ARVO Statement for the Use of Animals in Ophthalmic and Vision Research and was approved by the Vanderbilt University Animal Care and Use Committee. No procedures were performed that cause distress or suffering. Mice were provided food and water ad libitum and were monitored weekly by Vanderbilt University veterinary staff. When mice were anesthetized for procedures they were placed on a warming pad and monitored until they were awake and ambulatory. A total of 16 mice were used in this study. In the 7-month old cohort, 6 mice were injected with rAAV.eGFP and 5 mice were injected with rAAV.EpoR76E. In the 12-month old cohort, 5 mice were injected with rAAV.eGFP and 4 mice were injected with rAAV.EpoR76E.

### Recombinant adeno-associated virus (rAAV)

Human Epo cDNA was obtained from Origene (Rockville, MD) and cloned into pBSK (Agilent, Santa Clara, CA). Site-directed mutagenesis was performed to convert two nucleotides, resulting in the amino acid change of R76E (Agilent, Santa Clara, CA). The EpoR76E was subcloned into pAAV2, amplified in Stbl 3 cells (Life Technologies, Carlsbad, CA) and purified using an endotoxin free Mega prep kit (Qiagen, Valencia, CA). Both rAAV2/8.CMV.eGFP and rAAV2/8.CMV.EpoR76E were produced by the University of Pennsylvania vector core and quantified by RT-PCR using the following primers: BGH-PolyA forward TCTAGTTGCCAGCCATCTGTTGT, and BGH-PolyA reverse TGGGAGTGGCACCTTCCA. Mice were injected in the quadriceps with 1x10^9^ genome copies (gc) rAAV2/8.CMV.eGFP or rAAV2/8.CMV.EpoR76E at postnatal day 10 using a beveled Hamilton Syringe. Mice were assessed at 7 and 12 months of age and then collected for histological analysis.

### Optical coherence tomography (OCT)

Mice were anesthetized with ketamine/xylazine and imaged on the Bioptigen ultra-high resolution OCT system (Durham, NC). Outer nuclear layer (ONL) thickness was quantified manually at 0.2, 0.4, and 0.6 mm from the optic nerve head in both the nasal and temporal direction using digital calipers (Bioptigen).

### Electroretinogram (ERG)

Flash ERGs were performed according to previously published methods.[28] Briefly, for scotopic ERGs, mice were dark-adapted overnight, and then, prior to recording, their eyes were dilated with 1% tropicamide and they were anesthetized with 25/8/550mg/kg of ketamine/xylazine/urethane and placed on a heated mouse platform within the Ganzfeld dome of a Diagnosys LLC Espion Electrophysiology system (Lowell, MA). Mice were exposed to scotopic (0.1cd/m^2^) and mixed bright (500cd/m^2^) flashes as well as photopic conditions (30 cd/m^2^ on a white background). For scotopic flash intensities, the flash frequency was 1Hz with an inter sweep delay of 2sec. For mixed bright flash intensities, the flash frequency was 1Hz with an inter sweep delay of 1min. Amplitudes were measured from trough to peak.

### Hematocrit and EPO quantification

Hematocrit was measured by capillary centrifugation of tail vein blood at time of collection. Quantification of serum and eyecup EPO levels was determined using a Quantikine human EPO ELISA kit according to manufacturer’s instructions (R&D Systems; Minneapolis, MN). All samples were measured in duplicate.

### Immunohistochemistry (IHC)

Mice were euthanized, eyes were oriented by a nasal cautery mark and then enucleated and immersion preserved in 4% paraformaldehyde in phosphate buffered saline (PBS) for at least two hours. Eyes were cryo-protected in 30% sucrose at 4°C overnight, embedded in tissue freezing media (Triangle Biomedical, Durham, NC), and sectioned at 10 microns thick on a Microm HM550 cryostat (Thermo Scientific, Pittsburgh, PA). Sections were rinsed in PBS and blocked in 5% normal donkey serum in PBS containing 0.5% BSA and 1%Triton-X-100 (PBT) for 2 hours at room temperature followed by overnight incubation in primary antibody in PBT at 4°C. Primary antibodies used were: anti-rhodopsin (1:100, Abcam, Cambridge, MA); anti-glial fibrillary acidic protein (GFAP; 1:400, Dako, Carpinteria, CA); and anti-M- and anti-S- cone opsins (gift of Dr. Jeremy Nathans, Johns Hopkins University). Sections were then rinsed in PBS and incubated at room temperature for 1 hour in secondary antibody (donkey anti-mouse- or donkey anti-rabbit-Alexa; Life Technologies, Carlsbad, CA). Finally, sections were rinsed in PBS, mounted with Vectashield containing DAPI (Vector Labs, Burlingame, CA), and imaged on a Nikon Eclipse 80i microscope and Andor camera (Andor Technologies, South Windsor, CT).

### Statistics

Data collection was performed in a masked fashion. Statistical analysis of ONL thickness was performed by multiple t-test for each location from the optic nerve head in both treatment groups. Each age group was assessed separately. Statistical analysis of the ERG amax and bmax amplitudes was performed in Graphpad Prism software using a 2-wave ANOVA and Tukey post-hoc test.

## Results

### Elevation of hematocrit and EPO levels in the eye and serum after systemic treatment with rAAV.EpoR76E

We previously reported that the human ELISA kit is 1.43 fold less sensitive for EPO-R76E.[29] Taking this into consideration, we detected 0.2 ± 0.3 mU/ml EPO in the serum and 1. 7 ± 0.5 mU/ml in the eyecup of mice treated with rAAV.eGFP. In contrast, mice treated with rAAV. EpoR76E had 14.0 ± 4.9 mU/ml EPO in the serum and 56.7 ± 4.5 mU/ml EPO in the eyecup. This is in agreement with our previous studies also showing that EPO-R76E enters and accumulates in the eye.[29] In addition we detected an expected increase in hematocrit of mice treated with rAAV.EpoR76E as compared to mice that received rAAV.eGFP, 56 ± 11% and 44 ± 2%, respectively.

### Less ONL thinning in rAAV.EpoR76E treated mice

**Fig 1:**
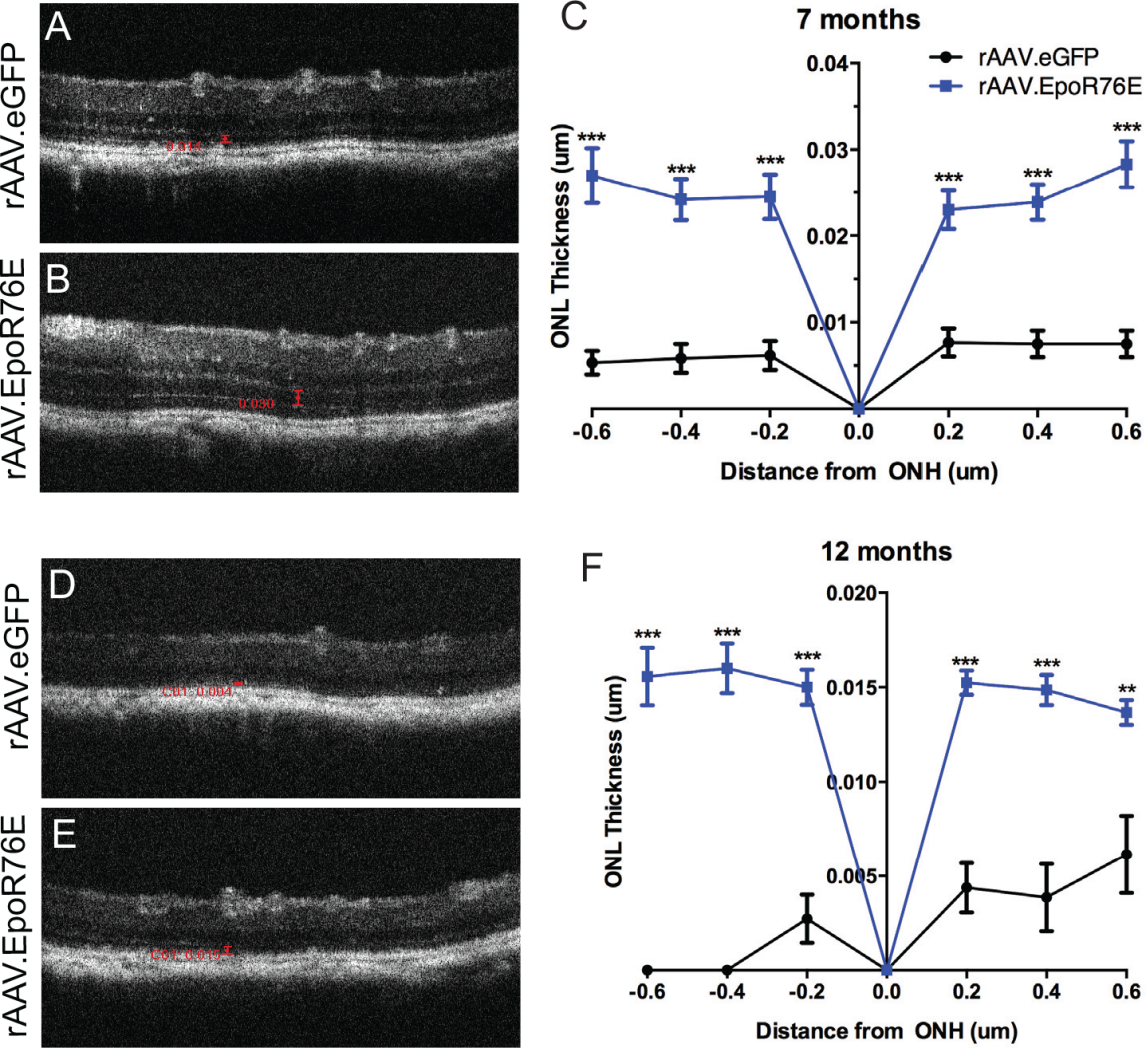
Systemic rAAV.EpoR76E preserves ONL thickness. A-C) OCT images (A, B) and quantification (C) in 7-month old hP23H *RHO*^+/-^,m*RHO*^+/+^ mice. D-F) OCT images (D,E) and quantification (F) in 12-month old hP23H *RHO*^+/-^,m*RHO*^+/+^ mice. Bars in images indicate ONL thickness. ***p<0.001.

The OCT images of hP23H *RHO*^+/-^,m*RHO*^+/+^ mice show a thicker ONL in mice injected with rAAV.EpoR76E as compared to mice injected with rAAV.eGFP at both 7 and 12 months of age (Fig 1A, 1B, 1D and 1E). A statistically significantly thicker retina was detected in the retinas from rAAV.EpoR76E treated mice at all locations assessed (Fig 1C and 1F; p<0.001). At 0.2 mm from the optic nerve the ONL thickness was 29 times thicker in the 7 month-old mice treated with rAAV.EpoR76E compared to those that received rAAV.eGFP (Fig 1C). The level of protection diminished at 12 months to 5 times thicker in the rAAV.EpoR76E treatment group as compared to rAAV.eGFP controls (Fig 1F). The continued thinning of the ONL despite treatment shows that EPO-R76E slowed the rate of degeneration, but did not block it altogether.

### rAAV.EpoR76E had a mild effect on visual function

**Fig 2:**
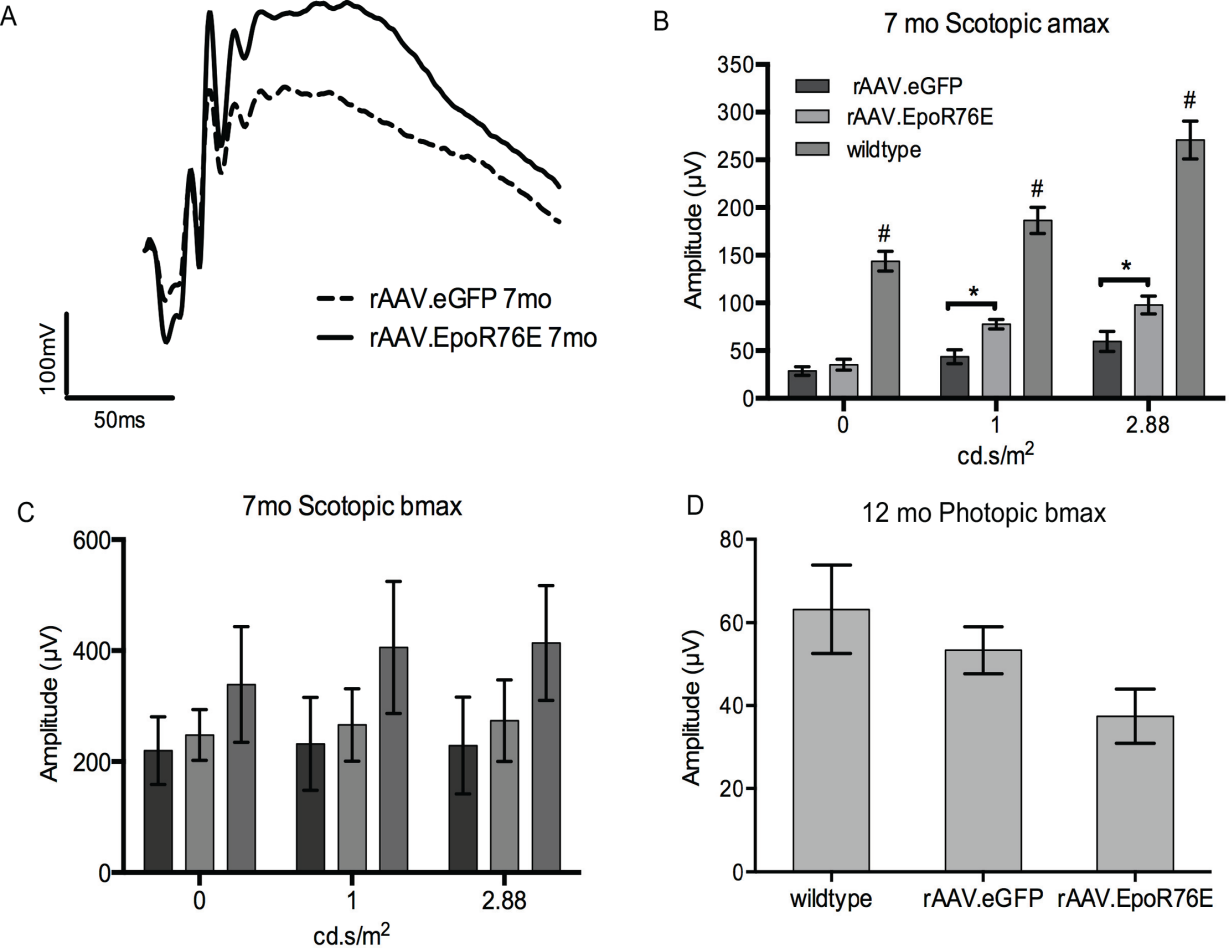
Systemic rAAV.EpoR76E preserves scotopic ERG amax. A) Representative waveforms from 7 month old hP23H *RHO*^+/-^,m*RHO*^+/+^ mice at 2.88 cd•s/m^2^. B) Quantification of the ERG amax at 0, 1, and 2.88 cd•s/m^2^ in wild-type and 7- month old hP23H *RHO*^+/-^,m*RHO*^+/+^ mice. ***p<0.001. C) Quantification of the scotopic ERG bmax in wild-type and 7-month old hP23H *RHO*^+/-^,m*RHO*^+/+^ mice. D) Quantification of photopic bmax in wild-type and 12-month old hP23H *RHO*^+/-^,m*RHO*^+/+^ mice.

Representative waveforms of 7 month old hP23H *RHO*^+/-^,m*RHO*^+/+^ mice treated with either rAAV.eGFP or rAAV.EpoR76E show larger a and b wave amplitudes in the rAAV.EpoR76E treated retina (Fig 2A). The scotopic *a* wave amplitude (amax) was higher in 7 month old hP23H *RHO*^+/-^ mice treated with rAAV.EpoR76E compared to those that were injected with rAAV.eGFP (Fig 2B; p<0.05). However, this difference was not apparent at 12 months of age (Fig 2B). The amax was also significantly reduced in the hP23H *RHO*^+/-^,m*RHO*^+/+^ mice at 7 months of age as compared to wild-type mice, regardless of treatment condition (Fig 2B, p<0.0001). There was also a trend for reduced scotopic b wave amplitude (bmax) in the hP23H *RHO*^+/-^,m*RHO*^+/+^ as compared to wild-type mice as a result of the decrease in the amax (Fig 2C). However, the decrease in bmax did not reach statistical significance. No preservation of cone function was detected in response to rAAV.EpoR76E. In fact there was a trend for a decrease in the photopic bmax in the rAAV.EpoR76E treatment group at 12 months of age relative to the rAAV.eGFP treated mice, but statistical significance was not reached (Fig 2D).

### Systemic gene delivery of EpoR76E had no effect on cone opsin localization or glial reactivity

**Fig 3:**
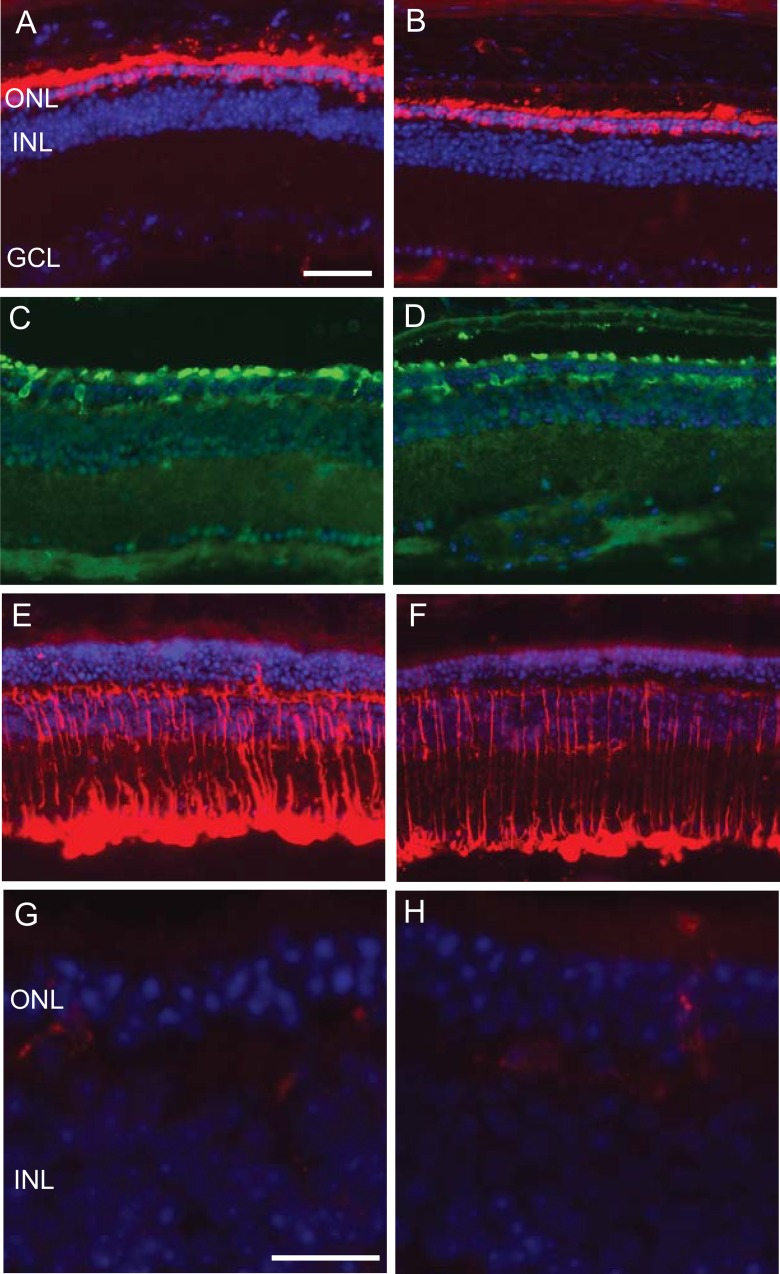
Systemic rAAV.EpoR76E has no effect on opsin localization or glial reactivity. Representative fluorescence micrographs near the optic nerve of 12-month old hP23H *RHO*^+/-^,m*RHO*^+/+^ mice treated with rAAV.eGFP (A, C, E), or rAAV.EpoR76E (B, D, F) labeled with DAPI (blue) and anti-rhodopsin (red; A, B), anti-M/L and anti-S-cone opsins (green; C,D), or anti-GFAP (red; E,F). Scale bar represents 50μm. Examples of microglial infiltration into the ONL in 12-month old hP23H *RHO*^+/-^,m*RHO*^+/+^ mice treated with rAAV.eGFP (G), or rAAV.EpoR76E (H), DAPI (blue), anti-IBA1 (red). Scale bar represents 25 μm.

There were regional differences in terms of retinal thickness in 12-month old hP23H *RHO*^+/-^ mice regardless of treatment indicating a non-uniformity of the retinal degeneration. This is demonstrated to some extent by comparing the ONL thickness in Fig 3A–3D to that in Fig 3E and 3F, which were taken slightly further away from the optic nerve head. For the representative fluorescence micrographs we chose regions with similar ONL thicknesses for each treatment group for a particular antibody. In regions where the ONL was thicker the outer segments were also longer, but the relative pattern was similar (data not shown). Representative micrographs from rAAV.eGFP (Fig 3A) and rAAV.EpoR76E (Fig 3B) treated 12-month old hP23H *RHO*^+/-^ mice show that rhodopsin localization was shifted to the plasma membrane of the photoreceptor cells in all mice, regardless of treatment or age. Similarly, representative micrographs from rAAV.eGFP (Fig 3C) and rAAV.EpoR76E (Fig 3D) treated 12-month old hP23H *RHO*^+/-^ mice also show cone opsin labeling in the remaining outer segments of all mice, regardless of treatment or age. Glial reactivity was assessed by immunolabeling with anti-GFAP to assess changes in the Muller cells, and anti-IBA1 to assess changes in the microglial cells (Fig 3G and 3H). Increased GFAP immunolabeling throughout the Müller cell processes was consistently detected across the retina in both rAAV.eGFP (Fig 3E) and rAAV.EpoR76E (Fig 3F) treated 12-month old hP23H *RHO*^+/-^ mice. Microglial cells, which are normally restricted to the inner retina, were occasionally detected in the ONL in both the rAAV.eGFP (Fig 3G) and rAAV.EpoR76E (Fig 3H) treated mice. Notably, microglia in the ONL was more common in areas of thinner retina.

## Discussion

The P23H mutation in rhodopsin is a Class II, i.e., misfolding, mutant that forms aggregates and interacts with the ubiquitin proteasome.[30, 31] It has recently been shown that these P23H rhodopsin aggregates lead to destabilization of the rod photoreceptor outer segment disc in two animal models.[32] This was confirmed in an elegant study, in which transgenic mice were used to illustrate that rhodopsin copy number influences outer segment size.[33] When Price and colleagues performed the study on a P23H transgenic mouse, the increase in copies of wild-type rhodopsin led to better outer segment preservation due to a correlative decrease in the amount of P23H rhodopsin in the outer segment. These results also agree with the exciting gene therapy studies demonstrating partial rescue of the hP23H *RHO*^+/-^,m*RHO*^+/+^ retinal degeneration by delivery of wild-type rhodopsin alone, without removal of the hP23H *RHO*.[8, 34]

This led us to test the hypothesis that EPO may provide neuroprotective support that could be used in conjunction with rhodopsin gene therapy to attain an even greater level of preservation than was achieved by rhodopsin gene therapy alone. The results of this study demonstrate that systemic gene delivery of EpoR76E slows the rate of photoreceptor cell death in hP23H *RHO*^+/-^,m*RHO*^+/+^ mice, but the efficacy decreases over time despite continued gene expression of EpoR76E.

Surprisingly, although P23H rhodopsin activates the unfolded protein response and ER stress, inhibiting the ER stress proteins, CHOP or ASK 1 is not sufficient to protect the photoreceptors. Thus, although EPO can block CHOP and other ER-stress induced proteins, that pathway was likely not involved in slowing photoreceptor cell death in this study.[15, 35, 36] Calpain, caspase, Bax, and cathepsin D are all activated in the P23H mutant rhodopsin retina. However, the increase in calpain correlates temporally most closely with DNA fragmentation, and inhibition of calpain slows photoreceptor cell death in the P23H rhodopsin mutant rat.[37–40] Treatment with EPO decreases calpain activity in other models of CNS injury/degeneration, so this could explain the neuroprotective effect detected in this study.[41, 42] It is well established that EPO can also block cell death by increasing levels of BclXL, which would counteract the increase in Bax induced in this model (for review see [13]). Finally, levels of autophagic proteins are increased in the P23H mutant rat and photoreceptor degeneration is inhibited by treating with the mTOR signaling inhibitor, rapamycin.[39] It is feasible that the photoreceptors ultimately succumbed to autophagic cell death regardless of EPO treatment. In summary, although EPO can block cell death pathways activated in the hP23H *RHO*^+/-^,m*RHO*^+/+^ retina, cell death still progressed potential due to an incomplete block of all possible cell death pathways.

In addition to these intrinsic pathways, ongoing glial reactivity may induce a toxic environment for the photoreceptors over time in the hP23H *RHO*^+/-^,m*RHO*^+/+^ mouse. An alternative neuroprotective approach to blocking cell death pathways would be to modulate glial reactivity. Intraocular delivery of EPO prevents an increase in GFAP in Müller cells in the *retinal degeneration slow* mouse.[16] However, in this study, consistent with our previous studies, systemic gene delivery of EPO-R76E did not decrease Müller cell reactivity. It is feasible that preventing reactivity of the Müller cells (as well as blocking photoreceptor cell death pathways) by delivering EPO directly into the eye may enhance neuroprotection in this model.

Protection of the cone photoreceptors is of greatest importance for patients since these cells are critical for high acuity vision that is used for much of our daily activities including seeing faces, reading, and driving. Since RP affects the rods first, peripheral vision is lost first. Cone degeneration and loss of central vision occur in end-stage disease after the loss of the rods. Treatment with EPO or EPO-R76E, particularly with an intraocular route, may extend the time frame of photoreceptor survival, potentially allowing patients to continue to have useful vision for a longer period of time. While we did not observe a protection of the photopic ERG response, we did observe a preservation of the outer nuclear layer in the posterior retina as measured by SD-OCT (Fig 1). Since full-field ERG represents the aggregate response of the whole retina, it is possible that regional protection is obscured when the full-field response is averaged. In the future, cone function could be measured in rAAV.EpoR76E treated animals by a light adapted behavioral assay such as the optokinetic response or the Morris water maze [43].
